# Early Onset Prosthetic Joint Infection and Bacteremia due to* Campylobacter fetus* Subspecies* fetus*

**DOI:** 10.1155/2017/5892846

**Published:** 2017-08-08

**Authors:** Igor Dumic, Mohan Sengodan, Joni J. Franson, Diego Zea, Poornima Ramanan

**Affiliations:** ^1^Department of Hospital Medicine, Mayo Clinic Health System, Eau Claire, WI, USA; ^2^Division of Clinical Microbiology, Mayo Clinic Health System, Eau Claire, WI, USA; ^3^Division of Infectious Diseases, Mayo Clinic Health System, Eau Claire, WI, USA; ^4^Division of Clinical Microbiology, Mayo Clinic, Rochester, MN, USA

## Abstract

*Campylobacter fetus* is a zoonotic pathogen that occasionally causes serious, relapsing, invasive disease, especially in immunocompromised hosts. We report a case of relapsing* C. fetus* diarrheal illness in a 75-year-old woman which resulted in secondary bacteremia and seeding of the left knee prosthetic joint. Patient responded favorably to debridement and retention of prosthesis in addition to six weeks of meropenem followed by chronic oral doxycycline suppressive therapy.

## 1. Introduction

*Campylobacter* prosthetic joint infection (PJI) is uncommon despite the increased occurrence of arthroplasty surgeries and food-borne campylobacteriosis.* C. fetus*, the pathogen responsible for the majority of published cases of* Campylobacter* PJI, has a propensity to cause invasive infection in elderly, immunocompromised hosts, particularly those with exposure to farm animals [1]. The need for special isolation techniques may preclude the early diagnosis and treatment of* C. fetus* gastrointestinal disease in high-risk patients, often leading to subsequent hematogenous dissemination and PJI [1]. As illustrated in our case, elective arthroplasty surgery should be postponed in high-risk patients who have had a recent episode of febrile diarrheal illness to prevent subsequent* C. fetus* PJI.

## 2. Case Report

A 75-year-old woman, who underwent elective left total knee arthroplasty one week prior to symptom onset, presented with acute onset of nausea, vomiting, diarrhea and worsening left knee pain of one day duration. Her medical history was significant for atrial fibrillation, pacemaker implantation for sinus node dysfunction, coronary artery disease, and insulin dependent type II diabetes mellitus. She lived on a farm by herself, denied illicit substance abuse, and had no recent travel. Three weeks prior to the left total knee arthroplasty, she was hospitalized with similar symptoms of nausea, vomiting, and diarrhea. At that time, patient was afebrile and clinically stable. Laboratory data had showed normal white blood cell (WBC) count, positive fecal leucocytes in stool, and negative cultures from blood and stool. She received symptomatic treatment with antiemetics and intravenous hydration and was discharged home the next day.

Patient was asymptomatic at the time of the left knee arthroplasty surgery which was done three weeks after the first hospitalization. She had an uneventful perioperative course and was discharged to a rehabilitation facility on postoperative day 3. However, patient was hospitalized again on postoperative day 7 due to recurrence of vomiting and diarrhea associated with worsening left knee pain. Patient denied fever, chills, and abdominal pain. Stools were loose, green in color, and without blood or mucous. On physical examination, she was afebrile with stable vital signs but was lethargic and weak. Abdomen was soft and nontender with normal bowel sounds. The left knee was tender and had a well healing surgical incision without surrounding erythema, wound dehiscence, or discharge. The rest of the physical examination was unremarkable. She had a WBC count of 6.3 × 10^∧^9/L with normal differential, hemoglobin of 9.4 g/dL, platelet count of 209 × 10^∧^9/L, serum creatinine of 1.23 mg/dL, lactate of 1.4 mmol/L, and an elevated C-reactive protein of 244.8 mg/dL. Computerized tomography of the abdomen and pelvis did not show any acute abnormalities. X-ray of the left knee did not show effusion or fracture.

Stool culture was repeated and resulted negative. Two sets of blood cultures from hospitalization days 1 and 3 signaled positive in the Bactec FX system (Becton-Dickinson, Sparks, MD, USA) within 48 to 72 hours in the aerobic bottles only (Bactec Plus Aerobic/F and Bactec Peds Plus/F vials). Gram stain showed faint staining, curved Gram negative bacilli. No targets were detected on the FilmArray Blood Culture Identification (BCID) panel (BioFire Diagnostics, LLC, Salt Lake City, UT, USA). After 24 hours of incubation of the positive blood culture broth in a microaerophilic environment, small, grey colonies were noted on the sheep blood agar and chocolate agar plates. The organism was identified as* Campylobacter fetus* subspecies* fetus* by matrix assisted laser desorption ionization time of flight mass spectrometry (MALDI-TOF MS) (Bruker Daltonics, Billerica, MA, USA). Patient was initially started on piperacillin-tazobactam on hospital day two, but this was later changed to meropenem. Transthoracic echocardiogram did not show any vegetation and transesophageal echocardiogram was declined by the patient. On hospital day 4, left knee synovial fluid cultures grew* C. fetus.* Patient underwent left knee irrigation and debridement with synovectomy and polyethylene exchange on hospital day 7. Three sets of intraoperative left knee tissue cultures grew* C. fetus* subspecies.* fetus* in sheep blood and chocolate agar plates after 48 hours. In vitro susceptibility testing was carried out using agar dilution method. The minimum inhibitory concentration (MIC) for ciprofloxacin was 1 *μ*g/ml, meropenem, 1 *μ*g/ml, gentamicin, 1 *μ*g/ml, doxycycline, 0.5 *μ*g/ml, and erythromycin, 2 *μ*g/ml.

A PubMed search using “*Campylobacter fetus*” and “prosthetic joint infection” dated December 15, 2016, yielded 15 case reports published in the past three decades (Table 1). All these patients were in their sixth decade of life or older and most had significant underlying medical comorbidities or immunocompromising conditions. Most of these patients had surgical intervention in addition to antimicrobial therapy. With the exception of two patients who died of an unrelated cause, all the others recovered from the infection at the time of last follow-up.

Our patient received six weeks of intravenous antibiotic therapy with meropenem, following which she was transitioned to chronic oral suppressive therapy with doxycycline. At the time of the last follow-up (3 months after completion of intravenous antibiotic therapy), there was no evidence of recurrence of infection.

## 3. Discussion

Members of the genus* Campylobacter* are Gram negative, curved, motile bacteria that are best known to cause food-borne diarrheal illness.* C. fetus* is one among 24 currently recognized species within this genus [11]. Human infection due to* C. fetus* is rare but may potentially be fatal, especially in the immunocompromised host [1, 12].

While the majority of gastrointestinal campylobacteriosis are caused by* C. jejuni* and* C. coli*,* C. fetus* is primarily associated with extraintestinal, systemic illness which may relapse or persist for years [1, 12].* C. fetus* subspecies* fetus* is responsible for most human infections, whereas subspecies* veneralis* (which causes bovine venereal campylobacteriosis) and* testudinum* (which is associated with exposure to reptiles or Asian dishes made with reptiles) have rarely been associated with human disease [12].

*C. fetus* is primarily a zoonotic pathogen; it colonizes the intestinal and/or genital tract of cattle and sheep, sometimes causing septic abortion in these animals [1]. Mode of transmission to humans is mainly through the consumption of food products derived from sheep and cattle such as raw or undercooked liver, meat, unpasteurized dairy products, or contaminated water [1]. Cross contamination of other food products such as fruits and vegetables may occur in the food preparation area or by using contaminated surface water for irrigation. Unlike* C. jejuni/C. coli,* poultry ingestion is not associated with infection since the higher body temperature of poultry birds is hostile to* C. fetus* [1]. Occupational exposure due to improper handling of animals by farmers, veterinarians, and abattoir workers has been reported [1]. A nosocomial outbreak of* C. fetus* meningitis in a neonatal intensive care unit suggests possible human-to-human transmission [13]. In addition, a recent outbreak among men who have sex with men in Montreal, Canada, suggests sexual transmission [14].

The clinical manifestations of* C. fetus* infection depends on the host immune status and the clinical syndrome. Healthy hosts may present with culture-negative diarrheal illness [15]. Systemic infections tend to occur in immunocompromised individuals (e.g., patients with human immunodeficiency virus (HIV) infection, malignancy, splenectomy, cardiac or liver disease, diabetes mellitus, alcoholism, and geriatric populations), patients with medical implant devices, pregnant women, and young healthy individuals with occupational exposure to live animals or abattoir work (indicating exposure to a higher organism burden) [1]. Our patient had multiple risk factors for systemic infection including her age, diabetic status, and residence in a farm.* C. fetus* typically enters the blood stream from the gastrointestinal tract in susceptible hosts [15]. Bacteremia is often preceded or accompanied by diarrhea, as seen in our case [15].

*C. fetus* can cause endovascular infections and secondary seeding may occur in almost any body site. Infections of nervous system, lungs, abdomen, and musculoskeletal system have been reported [1]. Infection in pregnancy is often associated with fetal loss, neonatal sepsis, and/or meningitis [1].* C. fetus* has a surface-layer (S-layer) which forms a capsule-like structure and helps the organism to evade host defense. This may explain its ability to cause a prolonged, persistent, relapsing infection for many years [1].

As seen in our patient,* C. fetus* is rarely isolated from stool culture despite the frequent occurrence of diarrhea preceding or accompanying bacteremia. This may be explained by the fact that most laboratories use selective media (which contain antibiotics) and a higher incubation temperature of 42°C to optimize growth of* C. jejuni* and* C. coli* (thermophilic species) while suppressing the growth of other fecal microbiota.* C. fetus* is susceptible to cephalosporins (used in some selective media) and grows variably at 42°C, thereby making its isolation from conventional stool culture difficult, and potentially causing underreporting of* C. fetus* diarrheal illness [11]. For instance, our laboratory uses a selective medium called Campy CVA agar (Becton-Dickinson) which contains cefoperazone (which is inhibitory to* C. fetus*) at an incubation temperature of 42°C.

If* C. fetus* infection is suspected (based on exposure history), the laboratory should be notified to perform alternative isolation techniques such as the use of less selective media, incubation at lower temperature (37°C) in a microaerophilic environment, and filtration techniques used to increase the yield of isolating* C. fetus* from stool culture [11]. The BioFire FilmArray Gastrointestinal panel (BioFire Diagnostics, Salt Lake City, UT) is able to detect* C. jejuni*,* C. coli,* and* C. upsaliensis* but not* C. fetus*.* C. fetus* has been isolated from commonly used aerobic blood culture bottles [11]. If a Gram negative, curved rod is seen from positive blood cultures, the broth should be subcultured to blood agar plate and incubated at 35 to 37°C in a microaerophilic environment to increase yield of isolation [11].

Treatment of* C. fetus* invasive infections requires parenteral therapy; however there are no guidelines for optimal antimicrobial therapy. Resistance to ciprofloxacin, doxycycline, and erythromycin has been previously reported [16]. Fluoroquinolone use was associated with higher mortality in patients with* C. fetus* bacteremia [16]. Among 111* Campylobacter fetus* subspecies* fetus* strains isolated from 1983 to 2000 in Quebec, Canada, all were susceptible to ampicillin, gentamicin, meropenem, and imipenem, with 90% minimal inhibitory concentrations of 4, 1, 0.12, and ≤0.06 *μ*g/ml, respectively [17]. Systemic infections have been treated with imipenem, meropenem, ampicillin, gentamicin, or chloramphenicol [17]. Our patient responded favorably to six weeks of meropenem and has been placed on lifelong doxycycline.

*C. fetus* is an underrecognized and underreported pathogen which is capable of causing severe, invasive, sometimes fatal infection in humans. It may present as a chronic, persistent, relapsing infection. Alternative culture isolation techniques (different from those used for* C. jejuni* isolation) are needed for optimal isolation of the organism from stool. Patients presenting with febrile illness and diarrhea and who have risk factors for* C. fetus* bacteremia (such as residence on a farm and immunosuppression) should be evaluated for invasive* C. fetus* infection prior to elective arthroplasty surgery.

## Figures and Tables

**Table 1:** Patient characteristics, clinical features, and treatment outcomes ofpublished cases of *Campylobacter fetus* prosthetic joint infection.

Case number/references	Year	Age in years/sex	Infected arthroplasty site	Comorbidities	Animal exposure	Time from surgery to onset of infection	Bacteremia	Surgical intervention	Therapy	Outcome
(1) [2]	1986	70 M	Hip	Alcoholism, cirrhosis	ND	ND	ND	Replacement	Colistin, gentamicin, penicillin, doxycycline	Recovered

(2) [3]	1993	75 M	Hip	CLL, chronic prednisone	ND	7 days	ND	Debridement and prosthetic retention	Imipenem + gentamicin (5 weeks), lifelong suppression with amoxicillin	Recovered

(3) [4]	1994	68 F	Hip	RA on prednisone	ND	Several years	ND	Bone biopsy and drainage of pus with prosthetic retention	Gentamicin, azithromycin, chloramphenicol (4 weeks)	Died from an unrelated cause

(4) [5]	2003	76 M	Knee	None	No	1 week	+	No	Ceftriaxone (6 weeks), Cefuroxime (9 months)	Recovered

(5) [6]	2005	72 M	Hip	Alcoholism	ND	11 months	+	Debridement and prosthetic retention	Ceftriaxone (3 weeks), Roxithromycin (3 months)	Recovered

(6) [7]	2005	72 M	Bilateral Knee	None	Yes, cattle	4 years	ND	Two-stage replacement	Gentamicin, rifampin, ciprofloxacin	Recovered

(7) [8]	2012	71 F	Knee	RA on rituximab and steroids, DM	ND	ND	−	ND	Clindamycin + Doxycycline (3 months)	Recovered

(8) [9]	2013	70 F	Knee	Cirrhosis	ND	ND	+	One-stage replacement	Amoxicillin-clavulanate (6 weeks), Ciprofloxacin + clindamycin	Died from an unrelated cause

(9) [9]	2013	78 F	Hip	None	ND	ND	−	Synovectomy, debridement, prosthetic retention	Clindamycin (8 weeks) + Gentamicin (2 weeks), lifelong suppression with amoxicillin	Recovered

(10) [9]	2013	88 M	Hip	Lung cancer	ND	ND	+	Not done	Clindamycin (4 weeks) + Gentamicin (1 week), lifelong suppression with clindamycin	Recovered

(11) [9]	2013	85 F	Hip	Cirrhosis	ND	ND	+	Not done	Amoxicillin (4 weeks) + Gentamicin (1 week), lifelong suppression with amoxicillin	Recovered

(12) [9]	2013	52 F	Knee	ND	ND	5 months	−	One-stage replacement	Amoxicillin (6 weeks) + Clindamycin (6 weeks)	Recovered

(13) [9]	2013	76 F	Knee	ND	ND	ND	−	Synovectomy, debridement, prosthetic retention	Clarithromycin (12 weeks) + Gentamicin (1 week)	Recovered

(14) [9]	2013	75 M	Hip	Renal transplant	ND	4 days	+	One-stage replacement	Clarithromycin (12 weeks) + Gentamicin (1 week)	Recovered

(15) [10]	2016	77 F	Knee	ND	ND	15 days	ND	ND	ND	Recovered

ND: not documented, M: male, F: female, CLL: chronic lymphocytic leukemia, RA: rheumatoid arthritis, and DM: diabetes mellitus.
